# Acceptability and feasibility of FAMS-T1D mHealth intervention to optimize self- and social regulation for emerging adults with type 1 diabetes

**DOI:** 10.1186/s40814-024-01497-1

**Published:** 2024-04-30

**Authors:** Lindsay S. Mayberry, Deborah J. Wiebe, Makenzie Parks, MaryJane S. Campbell, Aislinn B. Beam, Cynthia A. Berg

**Affiliations:** 1 Department of Medicine, Vanderbilt University Medical Center, Nashville, TN 37203 USA; 2 Department of Biomedical Informatics, Vanderbilt University Medical Center, Nashville, TN USA; 3 Department of Psychological Sciences and the Health Science Research Institute, University of California, Merced, CA USA; 4 Department of Psychology, University of Utah, Salt Lake City, UT USA

**Keywords:** Type 1 diabetes, Intervention, Social support, Family support, Mobile phone

## Abstract

**Background:**

Among emerging adults with type 1 diabetes (T1D), self-regulation and social regulation skills can help avoid high A1c and diabetes distress. FAMS (Family/friend Activation to Motivate Self-care) is mobile phone-delivered intervention that supports development of these skills and is efficacious among adults with type 2 diabetes. However, the acceptability and feasibility of the FAMS intervention among emerging adults with T1D are unknown.

**Methods:**

Therefore, we adapted FAMS for in a new disease context and developmental stage and then conducted a 3-month mixed-methods pre-post pilot study. Participants were emerging adults with T1D and a friend/family member enrolled as a support person (optional). Feasibility/acceptability outcomes and associated progression thresholds were recruitment (≥ 70% eligible emerging adults), retention (≥ 85%), intervention engagement (≥ 70%), and satisfaction (≥ 70%). We also collected qualitative feedback to determine if the intervention addressed relevant needs and explored changes in outcomes of interest (family/friend involvement, self-efficacy, self-management, distress, A1c).

**Results:**

Recruitment rates indicate recruitment of emerging adults with T1D (*n* = 30) and their support persons (*n* = 20) is feasible — 79% of emerging adults who screened as eligible enrolled and 70% of enrolled emerging adults invited a support person. Emerging adults completed 98% of coaching sessions, and response rates to automated text messages were median 85% IQR [68%, 90%]. Changes in selected measures for outcomes of interest were in expected directions suggesting sensitivity to changes occasioned by the intervention in a future evaluative trial. Emerging adults said FAMS-T1D helped with setting realistic goals, motivated them to prioritize diabetes goals, and increased support, indicating acceptability of the intervention in this new disease and developmental context.

**Conclusions:**

Findings suggest potential for FAMS-T1D to engage emerging adults and their support persons and feasibility for an evaluative trial examining effects on self-regulation (self-efficacy, self-management), social regulation (family/friend involvement), and outcomes (diabetes distress, A1c).

**Trial registration:**

We did not register this study on ClinicalTrials.gov because the purpose of the study was to assess the feasibility and acceptability of the intervention and study procedures and measures in preparation for a future trial. The purpose of that future trial will be to evaluate the effect of the intervention on health-related biomedical and behavioral outcomes, and that trial will be registered accordingly.

## Key messages regarding feasibility

1. What uncertainties existed regarding the feasibility?
  Would emerging adults with type 1 diabetes be willing to enroll in a study to evaluate the FAMS intervention?
  Would emerging adults with type 1 diabetes be willing to invite a support person to enroll, and would those invited support persons enroll?
  Would emerging adults with type 1 diabetes engage with the intervention and study assessment protocols sufficiently to support an evaluative study?
  Is there need for and/or perceived benefit of the FAMS intervention for emerging adults with type 1 diabetes?
  Were selected study measures sensitive to changes intended by the FAMS intervention, to be used in a subsequent randomized trial designed to evaluate effects on outcomes?
2. What are the key feasibility findings?
  Enrollment of emerging adults (79%) was high.
  Engagement of emerging adults with intervention components was high (≥ 85%), and completion of study assessments was high (≥ 93%).
  Satisfaction (93%) and usability (> 90th percentile) were high.
  Qualitative data indicated FAMS addressed relevant needs of emerging adults, which were consistent with the needs it was designed to address.
  Selected measures were sensitive to potential changes occasioned by the intervention and mirror qualitative findings on perceived benefits of the intervention.
3. What are the implications of the feasibility findings for the design of the main study?
  FAMS-T1D was extremely well-received by emerging adults with type 1 diabetes.
  Enrollment and study assessment completion rates were sufficient to support an evaluative trial.
  Selected measures are sensitive and likely to capture changes of the intervention relative to a control group in a future evaluative trial.

## Introduction

Approximately, three million Americans live with type 1 diabetes (T1D), and incidence is growing at an annual rate of 1.9% [1]. Early emerging adulthood (ages 18–24) is a high-risk time for T1D management [2]. Less than one-third of emerging adults complete self-management behaviors as recommended [3], only 17% meet glycemic targets, and during this developmental window, many experience their highest lifetime A1c [4] and elevated diabetes distress [5]. Longitudinal studies suggest that up to 50% of individuals with T1D experience the beginning of diabetes-related complications in their 20s, enhancing their risk for complications later in adulthood [6, 7] such as hypertension, kidney disease, and retinopathy [8].

Early emerging adulthood is a time of transition to more autonomy and changes to social contexts (e.g., school and work environments, moving out of the family home/hometown, transitioning to adult care). As a result, this is a challenging time for T1D management. Theoretical and empirical evidence indicates emerging adults are still developing the self-regulation skills to control their emotions, cognitions, and behaviors to manage T1D independently and consistently [9, 10]. As an example, T1D requires managing cognitions (remember to test blood glucose and focus on responding to values), emotions (minimize or override distress associated with the task or the resulting values), and behaviors (have and take insulin on time) across multiple changing environments and circumstances [10]. Emerging adults with poorer self-regulation skills (assessed by executive function tests) experience a more rapid increase in A1c across emerging adulthood (1.5% increase in HbA1c across ages 18–21) [11] and more daily self-regulation failures (e.g., forgetting or feeling unmotivated to monitor blood glucose) [12]. On a day-to-day basis, reporting more self-regulation failures is associated with lower self-management behaviors and fewer blood glucose checks [12]. Individuals with poorer self-regulation skills are less likely to plan specific, daily strategies to accomplish diabetes goals [13] and more likely to experience the occurrence of diabetes problems in daily life [14].

High-quality social support may offset deficits in self-regulation. Unfortunately, during this time of developing self-regulation skills, emerging adults also lack social support [9, 10]. Performing T1D self-management around new and changing friends, co-workers, and romantic partners may complicate self-regulation demands by adding emotional, social, or logistical barriers to navigate. This requires social regulation skills, such as asking for help needed, preventing others from interfering, and addressing others’ thoughts, feelings, or questions about T1D management. Social regulation skills may help augment gaps in self-regulation. For instance, disclosing information about diabetes activities to parents is more important for young adults with lower versus higher self-regulation skills [15]. In a daily diary study, when emerging adults could coordinate their self-regulation with social regulation, they experienced better A1c than when they are not engaged with others [16].

Social regulation includes skills to optimize social support *across relationships *by disclosing needs to others, soliciting help, and setting boundaries with others. There is increasing demand for social regulation skills during emerging adulthood for T1D for several reasons. First, parental involvement, which is beneficial for self-regulation [17, 18], declines across adolescence and emerging adulthood [15]. Parents are often perceived by emerging adults as critical of their T1D management [19]. Romantic partners and friends increasingly become potential sources of support but are not uniformly beneficial [20] as emerging adults may not know how to utilize them as sources of support or have concerns about “burdening” them [19]. Furthermore, emerging adulthood is marked by increases in new social relationships, such as co-workers, roommates, and friends, which place demands on the person with diabetes to disclose to others who are unfamiliar with T1D [21, 22]. Emerging adults are hesitant to disclose their T1D status to new friends and peers [19]; however, when trusted friends are aware of the emerging adult’s T1D and what to do in emergencies, friend support is beneficial [23].

Few high-quality interventions exist to address the unique needs of emerging adults with T1D [24]. Moreover, few interventions with emerging adults target social support [25] and the ones that do focus on increasing the people available through peer support groups [26, 27] rather than on developing social regulation skills to use across multiple social relationships encountered in daily life. FAMS (Family/friend Activation to Motivate Self-care) is a mobile phone-delivered intervention that helps set and support self-care goals and teaches skills to manage changing social relationships as well as to foster the relationship with a specific helpful support person (SP), when present. FAMS was developed with and for racially and socioeconomically diverse adults with type 2 diabetes (T2D) [28] and subsequently evaluated in a pilot randomized controlled trial [29]. Among adults with T2D, FAMS improved family/friend involvement by increasing helpful involvement and decreasing harmful involvement [29]. In addition, FAMS improved diabetes self-efficacy, diabetes self-care behaviors [29], and — among participants with elevated A1c at baseline — reduced A1c [29]. Therefore, we sought to adapt FAMS for emerging adults with T1D and examine its acceptability, feasibility, and pre-post changes among emerging adults with T1D in a pilot 3-month experience.

## Methods

Herein, we (a) explain the FAMS intervention’s core components and theoretical model, (b) describe processes to adapt FAMS for emerging adults with T1D, (c) describe the resulting FAMS-T1D intervention and (d) describe methods for the FAMS-T1D pilot study.

### FAMS intervention

This description of the FAMS intervention was unchanged in the adaptation for T1D. FAMS has three core components: (a) the person with diabetes (PWD) does structured monthly coaching to set SMART behavioral goals (specific, measurable, actionable, realistic, and time bound) and do skill building designed to elicit and manage family/friend involvement specific to that goal, (b) the PWD gets one-way and interactive text messages providing tailored goal support and monitoring, and (c) the option to invite a friend/family member to enroll as a SP to receive text messages designed to increase communication and autonomy supportive behaviors about diabetes and health goals [29]. FAMS does not require a SP’s involvement for participation [29] for two reasons. First, FAMS targets the PWD’s skills to regulate social relationships broadly, not just their relationship with an SP. Second, adults most in need of support for diabetes management and social regulation skills may not have a SP available and willing to enroll in a study.

Coaching sessions occur with the PWD alone (~25–30 min per session) and teach skills to be used with multiple friends/family members, not only the identified SP. Coaches do not interact with SPs. FAMS coaches are persons with clinical training through a master’s degree in clinical or counseling psychology or social work who are then trained in the FAMS coaching protocol. FAMS coaching combines Family Systems Theory [30–32] with basic health coaching, employing evidence-based techniques from goal setting theory [33], cognitive behavioral therapy (role-playing, homework) [34, 35], and health communication (teach-back) [36]. Each coaching session involves setting a PWD-directed behavioral goal and coach-selected exercise to build social regulation skills. Each session ends with an agreement to engage a specified other person in plans to meet the behavioral goal using skills learned in coaching (i.e., the “verbal contract”). Following the first coaching session, PWDs and their SPs start receiving text messages aimed at supporting themes discussed in coaching.

### Process to adapt FAMS for emerging adults with T1D

Existing FAMS coaching protocols and text message content were reviewed by experts in T1D management during emerging adulthood (“Acknowledgements”). Revisions were made to the text messages and the didactic sections of coaching protocols; specifically, goal-related psychoeducation was revised for T1D, and family/friend involvement-related psychoeducation was revised with examples specific to emerging adulthood. The coach who delivered all FAMS-T1D coaching was skilled and experienced in administering FAMS coaching to adults with T2D. She was trained in T1D-specific and emerging-adult-specific contexts by the same experts to enrich examples provided during skill building. The coach then practiced the revised coaching protocol with four young adults with T1D affiliated with the research team and elicited their feedback.

Next, we convened a stakeholder advisory board of *N* = 10 persons with T1D who participated in a prior longitudinal study of T1D through emerging adulthood and had indicated an interest in being contacted about future research. Stakeholder advisory board members were recruited from Texas (50%) and Utah (50%), 50% male and 40% racial or ethnic minority. Advisory board members completed a 1-h phone interview and reviewed revised text message content (~100 text messages each) via REDCap and provided feedback and suggested revisions for texts they did not like. Advisory board members were compensated US $25 for completing the phone interview and an additional US $25 per hour spent reviewing the text messages. Stakeholders were not eligible to participate in the subsequent pilot.

### FAMS-T1D

We developed a 3-month FAMS-T1D experience for both PWDs and SPs with our technology partner, PerfectServe, Inc. As in prior versions of FAMS, data from surveys and coaching sessions were entered into REDCap and sent via an application programming interface to our technology partner, who then sent tailored text messages and tracked PWD responses. PWDs received four monthly coaching sessions bookending 3 months of daily text messages.

#### FAMS-T1D coaching

For FAMS-T1D, personalized goals set during coaching were related to managing food (e.g., have a snack available when exercising 4 days/week), insulin (e.g., bolus 5 min before a meal for 7 days/week), and blood glucose (BG) monitoring (e.g., check BG before driving 6 days/week). The first coaching session included goal setting and a brief didactic section on the role of others in diabetes self-management, followed by homework to observe and note family/friend responses as PWDs worked to reach their goal. In addition to discussing goal progress and resetting the goal, each subsequent coaching session involved skill building to enhance social regulation, with the coach selecting the skill building activity best suited to address the PWD’s individual experiences. FAMS-T1D skill building activities included the following: activating supports, addressing resistance to involving others, assertive communication, collaborative problem solving, cognitive behavioral coping for goal failure, cognitive behavioral coping for harmful involvement, and developing an accountability partner. As described above, the skill building led to a verbal contract to implement the skill with a specific person in their life.

#### FAMS-T1D texts

Following each PWD’s first coaching session, PWDs and SPs began receiving text messages. Texts were tailored to participants’ preferred windows of time, names, goal set in coaching, and CGM use. Both PWDs and SPs received one-way and two-way (interactive) text messages, detailed below and in Table 1. PWDs received three or four one-way messages per week, either tailored to their goal or general content designed to support self- and social regulation. Monday through Saturday, PWDs received a goal assessment text around their bedtime asking them to report on their goal success for the day (i.e., “Did you meet your SMART goal today, Mon, 6/15? Please reply Yes or No”). Any response triggered encouraging automated feedback. Each Sunday, PWDs received a text prompting them to reflect on their goal progress and plans for the next week. The coach would read these texts biweekly and write a personalized response to their reflection. This interactive content was designed to support goal planning, monitoring, and motivation.

**Table 1:** FAMS-T1D message types, frequencies, and content examples

**Message type**	**Person with diabetes (PWD)**	**Support person (SP)**
One-way texts *tailored* to the PWD’s name and goal type and to the recipients’ preferred time of day	Sent 3–4 days per week (every other day). Some texts provide general information, tips, and encouragement for self-management and engaging friends/family in self-management support. Example text is as follows: “If you are having a hard time meeting your SMART goal, talking with a friend or family member might be helpful. Brainstorm together.” Some texts are tailored to self-management goal type. Example texts for BG monitoring goals are as follows: “Increasing your BG checking by even one check per day will help you manage your diabetes. You can meet your goal in small steps.”	Sent 3-4 days per week (every other day). Some texts provide general information, tips, and encouragement for providing support to the PWD. Example text is as follows: “Some of your support efforts might not go exactly the way you wanted, but don’t give up! Remember, Adam chose you for a reason!” Some texts are tailored to self-management goal type set by the PWD in coaching: example BG monitoring text is as follows: “Remembering to monitor BG throughout the day isn’t always easy. Ask Laura what you can do to help with her monitoring goal. You might find new ways!”
Interactive goal monitoring text *tailored* to the PWD’s goal and preferred time of day	Sent daily (Monday-Saturday) at PWD’s bedtime. Assesses achievement for PWDs’ personal self-management goal: “Did you meet your SMART goal today, Mon, 6/15? Please reply Yes or No” Automated feedback is sent upon text response. “Yes” triggers a response like the following: “Keep up the good work!” “No” triggers a response like the following: “Here’s a tip: If you know you have a busy day ahead of you, prepare the night before so you have your supplies when you need them. It helps to plan ahead.”	Not applicable for SPs
Interactive reflection text *tailored* to the PWD’s goal and the recipients’ preferred time of day	Sent once weekly on Sunday. Provides opportunity to reflect on progress and make a plan for next week: “This week is done! Your SMART goal was to _________________. What went well or got in the way? Reply with a brief reflection” PWDs who respond get automated feedback right away: “Thanks for this info! Your coach will reach out this week with some feedback.”	Sent once weekly on Sunday. Assesses support given to the PWD each week: “This week is done! Reflect on how you supported Jessica this week. Reply with what went well or what could go better next week” SP participants who respond get automated feedback right away: “Thanks for your response!”
Non automated message from coach	Sent once weekly on Monday in response to PWD responses to the goal assessment text. Coach-written text message response, which is specific to them and their goal and last coaching session. Examples of real coach-written messages are the following: • “Hi John, this your coach. You're right - planning almost always helps! I hope you at least had fun on your road trip. Keep up the good work!” • “Hi Sarah, this is your coach! Great job on staying consistent with recording. I bet your doctor will be happy to have some data to work with! Keep it up.”	Not applicable for SPs.

Enrolled SPs also received one-way and interactive text messages. Like one-way texts sent to PWDs, texts were either tailored to the PWDs’ goal or included general content designed to support dialogue about and autonomy support for T1D. Each Sunday, SPs received a text prompting them to reflect on their experiences supporting the PWD. Any response received automated feedback thanking them for responding.

### Pilot and feasibility study

#### Study design

We used a pre-post mixed-methods design to evaluate the 3-month FAMS-T1D experience among *N*= 30 PWDs and their SP (if enrolled). We selected this design and sample size because our goals were to determine if the adapted intervention would work well in new population and to test out recruitment and retention processes to ensure feasibility for a larger future trial [37].

Multiple data sources were used to examine feasibility [38] (i.e., recruitment and retention success) and acceptability [39] (i.e., engagement with intervention, satisfaction, usability, and perceived effectiveness with mixed methods). Surveys and A1c tests were administered at enrollment and post-intervention, and participants completed an exit interview after completing post-intervention data collection.

#### Recruitment and enrollment

Eligible PWDs were 18–24 years of age, diagnosed with T1D, taking insulin for ≥ 1 year, had a mobile phone, were comfortable sending texts, and could speak and read in English. Eligible SPs were at least 18 years old, had a mobile phone and were comfortable sending texts, and could speak and read in English. We excluded individuals with limitations that would preclude participation such as an intellectual disability, blindness or auditory limitations, or severe mental illness.

We recruited PWDs from the Utah Diabetes Endocrinology Clinic. We used electronic medical record data to identify potentially eligible PWDs seen in the clinic in the prior 6 months. We sent an opt-in/opt-out letter describing the study to potential participants before contacting them via call or text message to explain the study to those who did not opt-out and then confirming eligibility among those who expressed interest. PWDs who were eligible and interested were asked to identify a SP and communicate with their SP of choice to ask them to participate in the study. SPs willing to participate agreed to the PWD sending their contact information to the study team. The study team then contacted SPs via email or text to describe the study, confirm eligibility, and answer any questions. After individuals agreed to participate over phone or text, they were sent the consent form to sign via REDCap.

#### Data collection

We collected process data from recruitment and enrollment, pre- and post-surveys from PWDs and SPs, pre- and post-A1c tests from PWDs, intervention engagement data, and exit interviews with both PWDs and SPs to describe their experiences with FAMS-T1D. We also collected qualitative feedback indicating the intervention addressed relevant needs, examined process data, and examined changes in outcomes of interest (family/friend involvement, self-efficacy, self-management, distress, A1c).

#### Measures

Feasibility outcomes and associated progression thresholds were recruitment (≥ 70% eligible emerging adults) and retention (≥ 85%). Acceptability outcomes included intervention usability, intervention engagement, qualitative data and pre-post measures indicating perceived effectiveness, and retrospective satisfaction with the intervention and its components [39]. Thresholds for progression were intervention engagement (≥ 70%) and satisfaction (≥ 70%). Other measures would indicate more work would be necessary before progressing, such as a low usability score (< 85th percentile), lack of sensitivity of selected outcomes measures, and/or qualitative feedback indicating the intervention was not addressing relevant needs.

### Survey measures

Participants self-reported demographic information and diabetes characteristics (i.e., using CGM, using insulin pump, years since diagnosis). SPs reported on their relationship to the PWD and how far they lived from each other.

PWDs’ self-regulation was assessed with a measure of diabetes self-regulation failures and a measure of diabetes self-efficacy. Self-regulation failures were assessed with an 8-item measure developed among emerging adults with T1D to assess failures in emotional, cognitive, and behavioral control related to diabetes goals [12]. Example items include “Checking my blood glucose values kept slipping my mind” and “I was in a bad mood and didn’t really care about checking my blood glucose levels” with responses on a Likert scale from 1 = “strongly disagree” to 5 = “strongly agree.” Items were averaged such that higher scores indicate more self-regulation failures. Cronbach’s α was 0.91 (excellent) in our sample. Diabetes self-efficacy was assessed with the 10-item Self-Efficacy for Diabetes Management Scale [40], which asks respondents to rate their confidence that they can do various tasks on a scale from 1 = “not sure at all” to 10 = “completely sure.” Example tasks include “How sure are you that you can manage your diabetes even when you feel overwhelmed.” We added four items to capture transition issues common among emerging adults, such as “make your doctor’s appointments” and “deal with insurance” to the scale. Items were averaged such that higher scores indicated more confidence or self-efficacy managing T1D (*α* = 0.80, good).

PWDs’ social regulation was assessed with the Family/friends Involvement in Adults’ Diabetes (FIAD) [41]. The FIAD queries frequency of helpful (nine items, e.g., “How often do your friends or family members…exercise with you or ask you to exercise with them?”) and harmful (seven items; “…point out in front of others when you are eating unhealthy foods, like at a party or get-together?”) behaviors from family/friends over the prior month. Each score was obtained by summing responses on a scale from 1 = “never in the past month” to 5 = “twice or more each week” such that higher scores reflect more experience of helpful or harmful involvement, respectively. The FIAD was developed and validated among adults with T2D, so experts in T1D reviewed the items to ensure face validity for T1D. We adapted three items (e.g., “How often do you friends or family members…suggest you don’t need to check your blood glucose or take your insulin?” replacing “…suggest you don’t need to take your diabetes medicine?”). In our sample, Cronbach’s α was 0.86 (good) for helpful involvement and 0.93 (excellent) for harmful involvement.

PWDs’ outcomes of interest included self-management behaviors, diabetes distress, and hemoglobin A1c. Self-management behaviors were assessed with the Self-Care Inventory Revised [40] which is a 13-item measure assessing how often respondents perform T1D management behaviors such as checking blood glucose, administering insulin, eating healthfully, and exercising. Responses range from 1 = “never do it” to 5 = “always do this as recommended without fail” and are averaged such that higher scores indicate better self-management (*α*= 0.75, acceptable). PWDs’ diabetes distress was assessed with the Problem Areas In Diabetes (PAID) scale [42], a 20-item measure evaluating different dimensions of distress, including diabetes-related emotional problems, treatment-related problems, food-related problems, and social support-related problems. Response options range from 0 = “not a problem” to 4 = “serious problem” and are summed and transformed into a score ranging from 0 to 100, such that higher scores indicate more diabetes distress (*α*= 0.92, excellent). PWDs completed mail-in A1c kits provided and analyzed by CoreMedica Laboratories (Lee’s Summit, MO, USA) at enrollment and post-intervention. The kits have been validated against venipuncture and are preferred to venipuncture by patients [43].

SPs’ involvement was assessed with the family member version of the FIAD adapted for T1D, which asks the SP about their own behaviors (e.g., “How often do you…exercise with [PWD] or ask them to exercise with you?”). Cronbach’s α was 0.93 (excellent) for SPs’ self-report on helpful involvement, but 0.57 (poor) for self-report on harmful involvement — likely due to the small sample and low self-reported harmful involvement.

SPs’ desired involvement was assessed with two items from the DAWN Family Experience of Patient Involvement (DFEPI) [44]. Items ask how the SP feels about their current level of involvement relative to their desired level in the PWD’s “diabetes care” and in helping the PWD “deal with their feelings about diabetes.” We examined the percent of SPs reporting they were “as involved as they wanted to be” before and after the FAMS intervention to determine if FAMS increased alignment between SPs’ desired and actual involvement.

SPs’ diabetes distress was assessed with the PAID-5-DAWN Family Member [44] which asks how much the PWDs’ diabetes affects the SP. Like the PWD version, items are summed and transformed to a score ranging from 0 to 100 (*α* = 0.85, good).

SPs’ support burden was assessed with a single item from the DAWN2 study [44] to determine if FAMS changed support burden (with increased support burden being undesired). This item asks: “How much of a burden is it for you to help manage [PWD’s] diabetes?” with response options ranging from “no burden” to “a very large burden.” We examined mean change.

### Intervention engagement data

We used engagement data as part of our assessment of acceptability of FAMS-T1D. For coaching, we calculated the percent of completed sessions, and session components were tracked by the coach, including the following: the goal set during coaching, type of family/friend involvement discussed, the skill-building exercise employed, the verbal contract, and, for subsequent sessions, the outcome of the verbal contract from the previous session. PerfectServe, Inc. also tracked and shared data on participants’ response rates to the two-way text messages. Response rates were calculated as the number of two-way messages a participant responded to divided by the number of two-way messages they were sent.

### Exit interviews

Participants were invited to complete an exit interview after completion of study procedures. The exit interviews included the System Usability Scale (SUS) [45], the most widely used measure to assess usability of technology tools, which has established reliability, validity, and benchmark standards [46]. Respondents were asked 10 items about the ease of use, complexity, clarity, and integration of different components on a Likert scale (1 = “strongly disagree” to 5 = “strongly agree”). We tailored the items to FAMS-T1D, as the developers advise. The next section of the interview queried different components of FAMS-T1D using combinations of close-ended questions (e.g., “How often did you read the text messages we sent?” from 1 = “never” to 5 = “always”) and open-ended questions about experiences with FAMS-T1D (Table 3).

### Analyses

Descriptive statistics were used to characterize recruitment/enrollment, sample sociodemographic characteristics, participants’ intervention engagement, and participants’ feedback on quantitative items asked during the exit interview. We conducted paired *t*-tests to examine pre-post changes on continuous variables of interest and McNemar’s chi-squared test to examine pre-post changes in categorical variables of interest. The goal of these tests was to determine if selected measures were sensitive to the changes FAMS-T1D seeks to affect, not to evaluate effect sizes or test hypotheses, which is why we explored whether FAMS-T1D affected intended targets with mixed methods. Responses to open-ended interview questions were thematically coded to explore acceptability of FAMS-T1D components and changes experienced during the intervention. A coding team (“Acknowledgements”) developed thematic codes through an iterative process until codes were well-defined. Transcripts were coded using Dedoose software, with 46% coded by two coders to establish interrater reliability; disagreements were resolved through consensus. Cohen’s kappa ranged from 0.86 to 1.00, indicating strong intercoder reliability.

## Results

### Feasibility

Letters were sent to 86 potentially eligible PWDs; no one opted out. We contacted 46 by phone and screened 42 for eligibility. We enrolled 79% of those eligible with a weekly enrollment rate of 4.2 PWDs. All enrolled PWDs (*N* = 30) experienced the intervention and had the opportunity to invite a SP to enroll; 70% (*n* = 21) did so. All but one of the invited SPs (20/21) enrolled and participated. Nearly all PWD (97%; 29/30) and all SPs (100%; 20/20) completed a follow-up survey. Ninety-three percent (28/30) of PWDs and 100% (20/20) of SPs completed an exit interview.

### Sample characteristics

PWDs’ average age was 21.6 ± 1.4 years, 57% were female, 90% were non-Hispanic White, 80% were using a CGM, and 73% were using an insulin pump. Enrolled SPs were 38% parents, 33% spouses/co-residing romantic partners, 19% non-coresident romantic partners, 5% siblings, and 5% friends. Over half (53%) reported annual household income (including parents unless financially independent) < US $35,000. Using pre-determined cut-points for diabetes distress (≥ 40 when scaled 0–100), 27% of PWDs and 20% of SPs had elevated distress at baseline. PWDs had a mean baseline A1c of 8.2% (*SD* = 1.6) with IQR [7.5, 8.7%].

### FAMS-T1D acceptability

The FAMS-T1D intervention had mean SUS scores from both PWDs (87.1) and SPs (86.8) that were over the 90th percentile compared to other technology tools, reflecting excellent usability [46]. Furthermore, 93% (26/28) of interviewed PWDs and 80% (16/20) of SPs indicated agreement that FAMS-T1D would be a positive addition for other people like them.

#### Coaching engagement

Engagement with coaching was extremely high, with 98% of coaching sessions completed. PWDs rated their confidence high (≥ 7 on 1–10 scale) that they could meet their goal in 94% of sessions, suggesting goals were attainable. PWDs shared their experiences of family/friend involvement in coaching sessions, with 44% describing both helpful and harmful aspects, 49% helpful only, and 7% harmful only. The most frequently selected skills to address helpful/harmful involvement were collaborative problem-solving (40%), activating supports (34%), and assertive communication (14%). In 97% of sessions, PWDs agreed to the verbal contract (i.e., to practice the skill with a specific identified person), 96% rated their confidence to do so as high (≥ 7 on 1–10 scale), and 83% rated their confidence as high that doing so would increase their ability to meet their goal. At subsequent sessions, 89% reported they had done their verbal contract; of those, 96% said this led to a positive interaction, and 86% received the desired change in family/friend involvement.

#### Text message engagement

PWDs’ engagement with the interactive messages was high, median response rate of 85% IQR [68, 90%]. Weekly interactive reflection texts elicited responses such as the following: “Having someone who knew about my goal seemed to help me motivate to reach it and just being busy got in the way,” “Having my husband remind me to bolus was helpful. I thought it would be annoying but I found that it was pretty helpful,” and “It was helpful for me to communicate with loved ones about my goal so that they could help me achieve it. It's hard not being at home and being on a different schedule.”

SPs had lower engagement with the interactive text messages, median 50% IQR [27%, 66%], but when SPs responded to weekly interactive reflection texts, they did reflect on their role supporting the PWD. Examples include the following: “I could have definitely made more of an effort to encourage her. I do think things went fairly well this week,”and “I am noticing that the stress and anxiety caused by diabetes is probably the hardest thing about having it. Having to regulate food is hard but feeling hopeless is the hardest.”

#### Acceptability of text messages

Eighty-two percent (25/28) of interviewed PWDs and 95% (19/20) of SPs said they read FAMS-T1D text messages “always” or “almost always.” Most (85%) PWDs rated the text messages as high (≥ 7 on 1–10 scale) for helping them stay on track with managing diabetes. About half of the PWDs (54%) said the number of texts was just right (36% said too many texts; 11%, too few). However, there was almost universal approval for the coach-written feedback sent via text, with 93% of PWDs saying they wanted this feedback the same amount or more frequently. Similarly, 65% of SPs reported the number of texts they received was just right (30% said too many; 5%, too few). Sixty percent of SPs rated the text messages as high (≥ 7 on 1–10 scale) for improving their ability to support the PWD in their health goal.

### Perceived effectiveness: changes experienced during FAMS-T1D

Intervention targets and outcomes changed in the hypothesized direction during the intervention experience (Table 2). There were improvements in PWDs’ self-efficacy, self-management behaviors, and diabetes distress. There was pre-post change in A1c of −0.3%. There was also improvement in SP’s report of helpful involvement, and the percentage of SPs reporting alignment between their desired and actual involvement increased, with no change in support burden for SPs.

**Table 2:** FAMS-T1D 3-month pre-post differences in proposed outcomes and mediators

**Measure**	**Pre**	**Post**	**Difference**	***p*** **-value**
**PWDs’ self-regulation**				
Diabetes self-regulation failures	2.21	1.99	−0.22	0.130
Diabetes self-efficacy	**7.05**	**7.59**	**0.54**	**.013**
**PWDs’ social regulation**				
Helpful family/friend involvement	2.28	2.44	0.16	0.394
Harmful family/friend involvement	1.68	1.53	−0.15	0.289
**PWDs’ outcomes**				
Diabetes self-management	**3.31**	**3.55**	**0.24**	**.006**
Diabetes distress	**30.52**	**21.85**	−**8.67**	**.008**
A1c (%)	8.21	7.94	−0.27	.098
**SP’s involvement**				
Helpful involvement	**2.74**	**3.15**	**0.41**	**.020**
Harmful involvement	1.37	1.31	0.06	0.446
**SP’s outcomes**				
Diabetes distress	27.00	24.00	−3.0	0.124
**SP’s support burden**	0.45	0.50	0.05	0.716
**SP’s desired involvement** ^a^	**Pre**	**Post**	***χ*** ^**2**^	***p*** **-value**
How involved would you like to be in [PWD’s] diabetes care?			**8.00**	**.005**
% reporting “as involved as I am now”	**40%**	**80%**		
% reporting “somewhat more” or “more involved”	**60%**	**20%**		
How involved would you like to be in in helping [PWD] deal with their feelings about diabetes?				
% reporting “as involved as I am now”	35%	60%	3.57	.059
% reporting “somewhat more” or “more involved”	65%	40%		

Tests of difference are paired t-tests or ^a^McNemar’s chi-squared test. Bold indicates values meeting a priori significance level of *p* < .05. *PWD* person with diabetes, *SP *support person

In exit interviews, we asked PWDs about the FAMS-T1D coaching and text messages and how FAMS-T1D affected communication about diabetes with family and friends (Table 3). Seventy-nine percent (22/28) agreed that FAMS-T1D increased their ability to manage their diabetes-related goals. Nearly 70% (19/28) said they talked to their friends/family about their health more than before FAMS-T1D; none said they talked to them less. Eighty percent (16/20) of SPs agreed that FAMS-T1D increased their ability to support the PWD with diabetes-related goals or concerns.

**Table 3:** Exit interview questions regarding experiences with FAMS-T1D

Coaching:
• What did you think about the coaching?
• What was the most useful thing that you did in coaching?
• What was the least useful thing that you did in coaching
Text messages:
• What was your favorite thing about the text messages that you received?
• What was the worst thing about the text messages that you received?
• What did you think about receiving a follow-up message from your coach?
Effects on family/friend involvement:
• Did you talk to family members and friends about your health more, less, or about the same? Tell me about that.
• Did coaching, text messages, or both increase your ability to ask for support from family and friends? Tell me about that.
• While you were in the FAMS-T1D program, did your thoughts change about the role of family and friends in your health behavior or diabetes management? How so or why not?

Themes in response to open-ended interview questions (Table 3) which were reported by more than 10% of the sample are shown in Table 4 with select representative quotes. These findings reflected high acceptability of FAMS-T1D components and that FAMS-T1D improved intervention targets. PWDs indicated coaching helped them set realistic achievable goals in ways they had not previously experienced, and that text messages provided motivation, reminded them to make diabetes goals a priority, and held them accountable. PWDs explained how the intervention components appeared to work together to increase PWDs’ disclosure to and solicitation of support from family and friends.

**Table 4:** Themes and example quotes from exit interviews with PWDs

**Coaching: Helped PWD set realistic achievable goals while enhancing accountability and support**
*Helped set realistic and achievable goals in ways not previously experienced:* “I had a lot of goals and didn’t know where to start…The coaching really helped me sort out my goals and find realistic ones and just feel like I had a support system.” “I’ve never actually set so many goals with my endocrinologist…it was cool to have those coaching sessions and…have like a goal to work towards.” *Enhanced accountability and problem-solving:* “You knew you had someone that you needed to report back to. It made me much more cognizant of when things were helpful and when they weren’t.” “Just being able to talk just about situations and problems that I'm having related to diabetes. Being able to find necessary yet, I guess compromisable ways to approach it that will be achievable.” *Increased support:* “I loved how supportive [the coach] was with my goals…very uplifting and supportive of where I was at.”
**Text messages to PWD: Provided reminders, accountability, and motivation to make diabetes goals a priority**
*Reminder to prioritize diabetes goal:* “Having a daily text forces you to have it on your mind more often, and it just becomes a habit.” “I used them more as like a reminder. I wanted them sent around lunch…my goal was to dose and check my blood sugar at lunch time.” *Automated texts were supportive and motivating:* “They never felt pushy or negative…it was like 100% supportive.” “Just the kind of constant like motivation…you got this…keep on your goal.” *End of day texts held PWD accountable to diabetes goal:* “I knew I was going to be asked if I met the goal and that motivated me to actually meet the goal.” “Accountability at the end of the day. Like…did you do [your goal?]...I was kind of like oh, why didn’t I do it? This came up, this came up. How can I prevent that from happening?” *Texts from coach were personal and supportive:* “I really liked that…I wasn’t just getting messages from an automated thing.” “Reminder [that] I do have support. There are people wanting to help me and I’m capable of doing it.”
**FAMS-T1D increased openness to disclosing diabetes needs to family and friends and soliciting help**
*More open to disclosing and receiving help:* “It helped me realize they’re interested in helping me with it. It’s not like this big, scary thing that I have to be embarrassed about talking to people.” “I was more confident and willing to reach out to people and more open about it.” *Engaged with others in new ways:* “In the past, I would just ignore [annoying comments]. Over the past 3 months, I’ve been proactive about…explaining why what they said was annoying to me” “It forced me to have the conversation with [husband], ‘This is what I want you to help me with. This is how I want you to do it, but don’t be overwhelming or overbearing about it.’”
**Components of FAMS-T1D worked individually and in combination to enhance dialogue and support**
*Coaching showed value of asking for help and being held accountable to doing so:* “Talking to my coach showed me … there’s a lot of people out there that are willing and wanting to support me through this journey with diabetes.” “Being held accountable for it … She’d always ask me if I had talked to anyone else.” *Text messages to SP provided opportunities for support:* “Texts helped prompt the discussion…If you get a text while just hanging out, it gives you the opportunity to bring it up.” “The other party that was involved with it was getting text messages also. So, it was kind of – it wasn't just like a one-sided thing. It was on both sides.…the texts were really helpful in kind of keeping that conversation going to remind both parties. *Text messages and coaching worked in combination:* “Whenever I got a text…it helped bring me back to the goals I had set. When talking to my coach, usually I was told to maybe bring [my goal] up to my mom or somebody else. And so, I kind of pictured that as part of the goal…it just kind of came full circle.” “I might not particularly want to reach out too much because you might feel like ‘oh, your diabetes is like your thing to control.’ But then the texts were there as a reminder…some people were actually trying to help you and they can definitely support you. The coaching also helped…each session had a little homework…tell a couple of people about your goal…that was definitely helpful.”

Rare codes reported by < 10% of sample are not reported

## Discussion

We found evidence of feasibility and acceptability of the FAMS-T1D intervention among emerging adults with T1D and the friend or family member who enrolled as their SP. Emerging adults engaged in the intervention components, including completing nearly all assigned coaching sessions and having high engagement with the text messages. Although SPs responded to texts less frequently, they reported high engagement through reading the texts. As this was a pilot study, with a short pre-post design, the goal of examining changes in outcomes of interest is to determine if selected measures were sensitive to the behavioral, psychosocial, and relational changes that FAMS-T1D seeks to affect. The goal was not to test hypotheses, which would be inappropriate given the sample size and study design [37]; rather, we examined whether FAMS-T1D affected intervention targets with mixed methods assessing perceived effectiveness [39]. Qualitative data and pre-post changes in validated measures indicate FAMS-T1D successfully addressed needs for self-regulation (goal setting, monitoring, planning) and social regulation. PWDs indicated improvement in received helpful and harmful involvement from their wider social network of friends and family members. PWDs who invited SPs selected diverse relationships, but most SPs were parents or romantic partners, and all but one invited SP enrolled. The PWD-SP relationship displayed increased helpful SP involvement and increased alignment with SP’s desired involvement, without increased support burden.

Findings from qualitative interviews regarding the various components of FAMS-T1D are consistent with the idea that both self- and social-regulation are needed for T1D management. The intervention components worked together, coherently, to address these needs. For instance, coaching assisted PWDs to set SMART goals, and text support aided in goal monitoring and assisted with self-regulation (remembering, keeping goals in mind). Coaching to address helpful and harmful aspects of others’ involvement together with texts provided emerging adults with opportunities to explore how to optimize the support that they receive from their social network for diabetes management. Engagement of SPs via texts increased their helpful involvement. Findings suggest diverse relationships, from parents to new romantic partnerships, can be engaged via FAMS-T1D. This is important as both types of relationships have been described by emerging adults as most utilized for support during this developmental period [47].

The results should be considered in the context of some limitations. First, the sample of young adults participating in the study had A1c values that were closer to target A1cs than reported in other studies of young adults [2, 11], which may indicate that the emerging adults who enrolled in our pilot were already doing better with diabetes management than most. Moreover, our sample was predominantly non-Hispanic White, and many were using CGM and/or insulin pumps. It is an important priority to evaluate the intervention in more racially, ethnically, and socioeconomically diverse samples of emerging adults with T1D. On the other hand, FAMS was originally developed and tested among diverse adults with T2D where it also showed high acceptability in longer-term examinations [27, 28], and our multi-site stakeholder advisory board was more diverse than the sample from this single-site pilot. Future examinations of FAMS-T1D should include a more diverse sample from multiple sites. Finally, quantitive findings were pre-post without a control group for reference, and the intervention was delivered for only 3 months. This was appropriate given the prior efficacy trial in T2D and the primary goals here to assess feasibility and acceptability in a new disease context and developmental lifestage [36]. Given these promising findings, the next step is to evaluate FAMS-T1D in a randomized controlled trial among diverse emerging adults with T1D and their SPs to determing intervention effects over a longer period of time.

FAMS-T1D provided support for self- and social regulation at a developmental time that has been described as high-risk [10] and for which few evidence-based interventions exist [24]. Emerging adulthood is marked by transitions that increase variability in daily routines and physical environments, and changing insurances and health systems, which all contribute to risk for gaps in health care (e.g., missed visits, transition to adult care). These challenges together with the daily self-care tasks needed to manage T1D place significant demands on individuals’ self-regulation in the context of a changing social environment. FAMS-T1D is an entirely remote, mobile phone-delivered intervention that has high potential to reach this population and support these daily demands across multiple social contexts. FAMS-T1D is also consistent with the American Diabetes’ Association 2021 recommendations update to include systems that combine technology and coaching to support diabetes self-management. Few other such interventions are designed to address both individual and social aspect affecting self-management, which are so critical during early emerging adulthood. In summary, our findings indicate high potential for FAMS-T1D to engage and support early emerging adults with T1D should it prove efficacious in improving outcomes in a future trial.

## Data Availability

Data available upon request after publication. Intervention materials available per copyright agreements.
